# Two-Dimensional Direction-of-Arrival Fast Estimation of Multiple Signals with Matrix Completion Theory in Coprime Planar Array

**DOI:** 10.3390/s18061741

**Published:** 2018-05-28

**Authors:** Haiyun Xu, Yankui Zhang, Bin Ba, Daming Wang, Xiangzhi Li

**Affiliations:** National Digital System Engineering and Technological Research R&D Center, Zhengzhou 450001, China; xuhaiyun1995@163.com (H.X.); zhang_yk_2018@163.com (Y.Z.); wdm_wangdaming@163.com (D.W.); lixiangzhi2231@163.com (X.L.)

**Keywords:** direction-of-arrival, coprime planar array, matrix completion theory, degree of freedom

## Abstract

In estimating the two-dimensional (2D) direction-of-arrival (DOA) using a coprime planar array, the main issues are the high complexity of spectral peak search and the limited degree of freedom imposed by the number of sensors. In this paper, we present an algorithm based on the matrix completion theory in coprime planar array that reduces the computational complexity and obtains a high degree of freedom. The algorithm first analyzes the covariance matrix of received signals to estimate the covariance matrix of a virtual uniform rectangular array, which has the same aperture as the coprime planar array. Matrix completion theory is then applied to estimate the missing elements of the virtual array covariance matrix. Finally, a closed-form DOA solution is obtained using the unitary estimation signal parameters via rotational invariance techniques (Unitary-ESPRIT). Simulation results show that the proposed algorithm has a high degree of freedom, enabling the estimation of more signal DOAs than the number of sensors. The proposed algorithm has reduced computational complexity because the spectral peak search is replaced by Unitary-ESPRIT, but attains similarly high levels accuracy to those of the 2D multiple signal classification algorithm.

## 1. Introduction

Direction-of-arrival (DOA) estimation is a significant problem in many applications, such as radar [1], underwater acoustics [2], indoor navigation, and so on [3]. At present, most existing DOA techniques use the uniform and non-sparse arrays, including uniform line arrays (ULAs) [4], uniform rectangular arrays (URAs) [5] and uniform L-shaped arrays [6]. These require the distance between the nearest sensors to be no more than half the wavelength of the impinging signals, and use high-resolution subspace algorithms [7,8,9,10] to obtain accurate parameters. With the development of technology, the requirement for more precise location determination requires arrays with more sensors and expanded apertures. Unfortunately, this makes the systems more complicated, and enhances the antenna mutual coupling interference, which increases the estimation errors.

To acquire both high array degree of freedom and the required precision, researchers have begun to examine the signal features. For example, the array aperture has been expanded based on non-circular signals, whose ellipse covariance is non-zero [11], and a virtual array has been constructed in the frequency domain based on orthogonal frequency division multiplexing systems [12]. The algorithm in [12] not only improves the precision of parameters, but also estimates multiple targets (more than the number of sensors). Although the above studies make the best use of information, they are confined by the application environment. Hence, the sparse and non-uniform array structure was proposed. The nested array in [13], which has a high freedom degree and precision, consists of a uniform and sparse array, and a uniform and non-sparse array. However, the nested array cannot avoid the mutual coupling interference among the sensors of the non-sparse array. Thus, a one-dimensional coprime array consisting of two uniform sparse line arrays was introduced in [14]. This weakens the mutual coupling interference, and simultaneously constructs a larger aperture with fewer sensors when compared with uniform and non-sparse arrays. The larger aperture obtains more precise estimates, and the M+N−1 sensors can achieve O(MN) degrees of freedom. Recently, two-dimensional (2D) coprime arrays and the corresponding DOA algorithms have been proposed [15,16,17]. The coprime planar array investigated in [15] has a complicated structure that could only be solved using the multiple signal classification (MUSIC) algorithm. As MUSIC uses a spectral peak search, the increase in accuracy is computationally expensive. A low complexity algorithm [16] has been applied to sparse L-shaped arrays. Although the number of signals that can be identified is limited to the number of sensors in these methods, parallel coprime subarrays have the ability to resolve $\left(M^{2}-1\right)/8$ signals with M sensors [17]. We will show that the proposed coprime planar arrays offer a higher degree of freedom than parallel coprime subarrays for the same number of sensors.

For DOA estimation using coprime planar arrays, the problems of high computational complexity and the inadequate array degree of freedom are overcome using an algorithm based on matrix completion theory. The method reconstructs the covariance matrix of the virtual uniform rectangular array, which is the same size as the coprime planar array, after analyzing the received signals’ covariance matrix. We apply the matrix completion theory to estimate the missing elements of the covariance matrix. Using the unitary estimation signal parameters via rotational invariance techniques (Unitary-ESPRIT), we then realize the fast estimation of the 2D DOA angle. This algorithm ensures high precise estimation values, as it maintains the same aperture and avoids the spectral peak search, effectively reducing the complexity. The most important point is that increasing the number of virtual sensors enhances the degree of freedom, which allows us to find more signals than with existing methods.

The remainder of this paper is arranged as follows. Section 2 introduces the model of coprime planar array model, and Section 3 describes the steps of the algorithm. Section 4 and Section 5 analyze the computational complexity and performance of the model, respectively, to demonstrate the validity of this algorithm. Section 6 gives the conclusion to this paper. 

The notations used in this paper are as follows: $I_{N}$ represents the N dimensional unit array; ${\left(•\right)}^{T}$ and ${\left(•\right)}^{H}$ respectively represent transposition and the conjugate transpose, respectively; E(•) denotes the mathematical expectation; diag(•) expresses the transformation of a vector to a diagonal matrix. 

## 2. System Model

**Figure 1:**
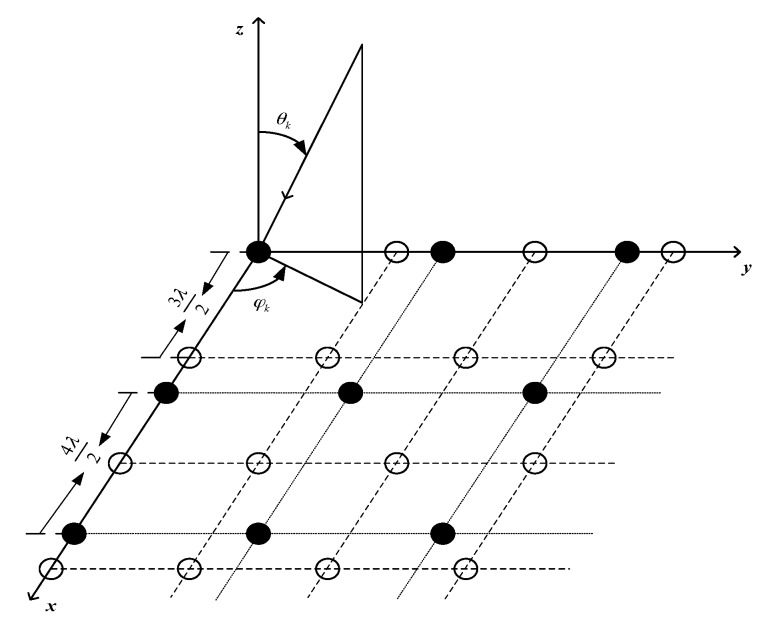
Geometry of coprime planar array when *M*_1_ = 3, *M*_2_ = 4.

Considering the coprime planar array model introduced in [15], the array geometry is shown in Figure 1. The coprime planar array is made up of two URAs. Subarray 1 has $M_{1}\times M_{1}$ sensors and subarray 2 has $M_{2}\times M_{2}$ sensors, where $M_{1}$ and $M_{2}$ are the coprime integers (generally assuming $M_{1}<M_{2}$) and denote the sensor numbers on the x,y axis. Correspondingly, the distance between the closest sensors is $M_{2}\lambda /2$ and $M_{1}\lambda /2$, respectively, where the λ represents the wavelength of the impinging signals. The subarrays coincide at the origin, so the total number of sensors is $M=M_{1}^{2}+M_{2}^{2}-1$. Suppose that there are K uncorrelated narrowband far-field signals impinging on the array with the power $\left\{\sigma_{1}^{2},\sigma_{2}^{2},\cdots \sigma_{K}^{2}\right\}$. The kth signal is located at elevation angle $\theta_{k}$, which is downward from the z-axis, and azimuth angle $\varphi_{k}$, which is counterclockwise from the *x*-axis. 

We define the $D_{1}$ and $D_{2}$ as the location set of the subarrays. $D_{1}=L_{1}\lambda /2$ and $D_{2}=L_{2}\lambda /2$, where the $L_{1}=\left\{\left(m,n\right)M_{2},0\leq m,n\leq M_{1}-1\right\}$ and $L_{2}=\left\{\left(m,n\right)M_{1},0\leq m,n\leq M_{2}-1\right\}$. Hence, the location set of the coprime planar array is expressed as $D=D_{1}\cup D_{2}$ and we have $L=L_{1}\cup L_{2}$. The received signals at the array can be represented as

(1) X(t)=A(φ,θ)S(t)+N(t)

The array manifold

(2) $$A\left(\varphi ,\theta \right)=\left[\begin{array}{cccc} a\left(\varphi_{1},\theta_{1}\right) & a\left(\varphi_{2},\theta_{2}\right) & \cdots & a\left(\varphi_{K},\theta_{K}\right) \end{array}\right],$$

where

(3) $$a\left(\varphi_{k},\theta_{k}\right)={\left[\begin{array}{cccc} a_{1}\left(\varphi_{k},\theta_{k}\right) & a_{2}\left(\varphi_{k},\theta_{k}\right) & \cdots & a_{M}\left(\varphi_{k},\theta_{k}\right) \end{array}\right]}^{T}$$

(4) $$a_{m}\left(\varphi_{k},\theta_{k}\right)=e^{j\pi \sin\theta_{k}\left(x_{m}\cos\varphi_{k}+y_{m}\sin\varphi_{k}\right)},\left(x_{m},y_{m}\right)\in L$$

The signal data vector

(5) $$S\left(t\right)={\left[\begin{array}{cccc} s_{1}\left(t\right) & s_{2}\left(t\right) & \cdots & s_{K}\left(t\right) \end{array}\right]}^{T},$$

where t=1,2,⋯J is the sampling time and J is the number of snapshots. And the noise vector

(6) $$N\left(t\right)={\left[\begin{array}{cccc} n_{1}\left(t\right) & n_{2}\left(t\right) & \cdots & n_{M}\left(t\right) \end{array}\right]}^{T},$$

where the elements are usually Gaussian random variables with zero means and variance $\sigma_{n}^{2}$.

## 3. The Proposed Algorithm

In [15], the DOA is estimated based on the two subarrays. Each subarray uses the subspace algorithm, but the results have some phase ambiguity. The two sets of results, including this ambiguity, have common values, and these are the real values. Therefore, the coprime planar array can be used to find DOAs. A partial spectral search (PSS) method is used to reduce the complexity caused by 2D-MUSIC mentioned in [15], but this does not significantly reduce the complexity.

### 3.1. Reconstructing the Covariance Matrix of the Virtual Array

Considering (1), the covariance matrix of the received signal is defined as

(7) $$R_{X}=E\left[XX^{H}\right]=AR_{S}A^{H}+\sigma_{n}^{2}I_{M},$$

where $R_{S}=diag\left(\begin{array}{cccc} \sigma_{1}^{2} & \sigma_{2}^{2} & \cdots & \sigma_{K}^{2} \end{array}\right)$. We define $R=AR_{S}A^{H}$ and express the element in the mth row and nth column as

(8) $$R_{m,n}=\sum_{k=1}^{K}\sigma_{k}^{2}e^{j\pi \sin\theta_{k}\left(\left(x_{m}-x_{n}\right)\cos\varphi_{k}+\left(y_{m}-y_{n}\right)\sin\varphi_{k}\right)}=R\left(x_{m}-x_{n},y_{m}-y_{n}\right),$$

where the $\left(x_{m},y_{m}\right)$ is one element of set L. According to [18], the covariance matrix of a uniform array can be estimated by that of a non-uniform array containing some of the same sensors. Consider a V×V URA, where $V=M_{1}M_{2}-M_{1}+1$. This is the same size as the coprime planar array. And we define the set as $\tilde{L}=\left\{\left(m,n\right),0\leq m,n\leq V-1\right\}$. Under the same situations, the covariance matrix without the noise is defined as $\tilde{R}$, and the element in the mth row and th column is expressed as

(9) $${\tilde{R}}_{m,n}=\sum_{k=1}^{K}\sigma_{k}^{2}e^{j\pi \sin\theta_{k}\left(\left(x_{m}-x_{n}\right)\cos\varphi_{k}+\left(y_{m}-y_{n}\right)\sin\varphi_{k}\right)}=\tilde{R}\left(x_{m}-x_{n},y_{m}-y_{n}\right),$$

where the $\left(x_{m},y_{m}\right)$ is one element of set $\tilde{L}$. Thus, we define the two sets as

(10) $$R_{Diff}=\left\{\left(x_{m}-x_{n},y_{m}-y_{n}\right)|\left(x_{m},y_{m}\right),\left(x_{n},y_{n}\right)\in L\right\}$$

(11) $${\tilde{R}}_{Diff}=\left\{\left(x_{m}-x_{n},y_{m}-y_{n}\right)|\left(x_{m},y_{m}\right),\left(x_{n},y_{n}\right)\in \tilde{L}\right\}$$

**Figure 2:**
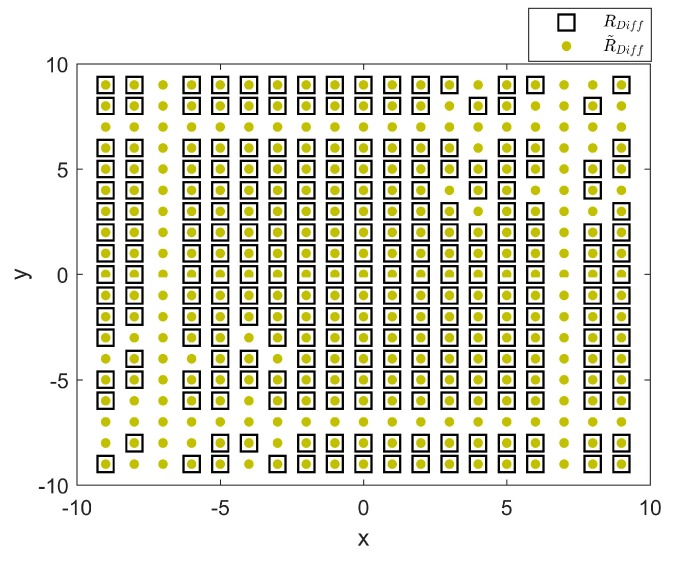
Values of sets $R_{Diff}$ and ${\tilde{R}}_{Diff}$ when $M_{1}=3,M_{2}=4$.

For $M_{1}=3$ and $M_{2}=4$, Figure 2 shows the values in $R_{Diff}$ and ${\tilde{R}}_{Diff}$. The figure shows that all elements of $R_{Diff}$ are included in ${\tilde{R}}_{Diff}$. Accordingly, we can use R to estimate $\tilde{R}$. However, some elements of ${\tilde{R}}_{Diff}$ do not exist in $R_{Diff}$. If we reduce the aperture of the uniform array, we can estimate all elements, with some loss of accuracy. As a result, we estimate $\tilde{R}$ with the values of R and tentatively set the uncertain values to zeros. 

In practice, the theoretical covariance matrix given by (7) is unavailable because the number of snapshots is limited. Instead, it is usually estimated as

(12) $${\overset{\wedge }{R}}_{X}=\frac{1}{J}XX^{H}$$

Hence, ${\overset{\wedge }{R}}_{X}$ replaces R and the estimated covariance matrix of the virtual URA is defined as ${\overset{\wedge }{R}}_{V}$.

### 3.2. Matrix Completion Theory

It has been found that the virtual ULA transformed from the one-dimensional coprime array has holes [19,20], but these assertions used convex optimization to estimate the zero-location values in the covariance matrix of virtual array under the condition that the hole values were zeros. Similarly, the matrix completion theory [21] can be introduced to estimate the zeros of ${\overset{\wedge }{R}}_{V}$. 

Firstly, the problem is rewritten as

(13) $$\begin{array}{l} \begin{array}{cc} \min & rank\left(R_{C}\right) \end{array}\\ \begin{array}{cc} s.t & P_{\Omega } \end{array}\left(R_{C}\right)=P_{\Omega }\left({\overset{\wedge }{R}}_{V}\right) \end{array},$$

where $R_{C}$ is the target matrix and $P_{\Omega }$ is the sampling operator. The values of $P_{\Omega }$ are denoted as the sampling location of one matrix. If $P_{\Omega }=\left\{\left(m-1\right)V+n,1\leq m,n\leq V\right\}$, we can set the values in the mth row and the nth column of a V×V target matrix (initializing the target matrix as null matrix) as that in same location of matrix operated. Once the structure of the coprime planar array has been determined, the location of the non-zero elements, represented by $P_{\Omega }$, is uniquely determined. As $R_{C}$ is low rank, we estimate the zero-location values by requiring the rank of the target matrix to be as low as possible. Solving the rank minimization is NP-hard, so we need to employ a convex relaxation method. Singular value thresholding (SVT) [21,22] is a computationally effective method that offers fast convergence. Given [22], SVT is a nuclear norm minimization approach and converts (13) to

(14) $$\begin{array}{l} \min\mu {\left\|R_{C}\right\|}_{*}+\frac{1}{2}{\left\|R_{C}\right\|}_{F}^{2}\\ s.t.{\left\|P_{\Omega }\left(R_{C}\right)-{\overset{\wedge }{R}}_{V}\right\|}^{2}\leq \varepsilon_{0} \end{array},$$

where μ is a constant, $\varepsilon_{0}$ is a constraint on the noise power, and ${\left\|R_{C}\right\|}_{*}=\sum_{i=1}^{n}\lambda_{i}\left(R_{C}\right)$, where $\lambda_{i}\left(R_{C}\right)$ denotes the ith singular value of $R_{C}$. The steps of the SVT algorithm are presented as follows:

**Step 1**: Choose appropriate values of μ,δ,ε and then initialize $R_{S}^{0}={\overset{\wedge }{R}}_{V}$;
**Step 2**: Calculate the $R_{C}^{i}=F_{\mu }\left(R_{S}^{i-1}\right)$ on the ith iteration;
**Step 3**: Update $R_{S}^{i}=R_{S}^{i-1}+\delta ({\overset{\wedge }{R}}_{V}-P_{\Omega }^{}(R_{C}^{i}))$;
**Step 4**: If ${\left\|P_{\Omega }\left(R_{C}^{i}\right)-{\overset{\wedge }{R}}_{V}\right\|}_{F}^{2}/{\left\|{\overset{\wedge }{R}}_{V}\right\|}_{F}^{}>\varepsilon$, let i=i+1 and return to step 2. If not, define $R_{C}=R_{C}^{i}$.

We call $F_{\mu }\left(R_{S}\right)$ a soft threshold operator. This is defined as

(15) $$F_{\mu }\left(R_{S}\right)=UF(\sum )V^{H},$$

where $R_{S}=U\sum V^{H}$; $\sum =diag\left(\begin{array}{ccc} \lambda_{1} & \cdots & \lambda_{M} \end{array}\right)$ and $\lambda_{i}$ represents ith singular value arranged from low to high; $F\left(\sum \right)=diag\left(\begin{array}{ccc} {\tilde{\lambda }}_{1} & \cdots & {\tilde{\lambda }}_{M} \end{array}\right)$, where

(16) $${\tilde{\lambda }}_{i}=\{\begin{array}{c} \lambda_{i}-\mu \begin{array}{cc} & \lambda_{i}\geq \mu \end{array}\\ 0\begin{array}{cc} & \lambda_{i}<\mu \end{array} \end{array}$$

In [22], the values of μ,δ,ε are considered; we set them as $\mu =5{\left(M_{1}M_{2}-M_{1}+1\right)}^{2}$, δ=1.21 and $\varepsilon =10^{-3}$, respectively. Finally, we obtain the covariance matrix of the virtual array by estimating the zero-location value of.

Before estimating DOAs, we need to know the number of signals, which is essential for accurate DOA estimation. Hence, we assume that the signal number is known. However, a method to detect more signals than the number of sensors is presented in [23,24], and the rank of matrix estimated by the algorithms is equal to the number of signals. All three methods aim to estimate a low rank matrix based on nuclear norm minimization. But the proposed algorithm is applied to a more complicated model, where the results are not only affected by the noise, but also by zero-value elements of ${\overset{\wedge }{R}}_{V}$. In spite of this, we can still use matrix completion to estimate the number of signals, and it would we worthwhile to analyze the relationship between the number of signals and the rank of $R_{C}$ in further research. 

Generally, we apply the 2D-MUSIC algorithm to find the azimuth and elevation angle of signals. From [25], the spatial spectrum function of angles is given by

(17) $$P\left(\varphi ,\theta \right)={\left\|U_{n}^{H}\tilde{a}\left(\varphi ,\theta \right)\right\|}_{2}^{-2},$$

where $U_{n}$ is the noise subspace of $R_{C}$ given by eigenvalue decomposition. $\tilde{a}\left(\varphi ,\theta \right)$ is expressed as

(18) $$\tilde{a}\left(\varphi ,\theta \right)={\left[\begin{array}{cccc} {\tilde{a}}_{1}\left(\varphi ,\theta \right) & {\tilde{a}}_{2}\left(\varphi ,\theta \right) & \cdots & {\tilde{a}}_{V^{2}}\left(\varphi ,\theta \right) \end{array}\right]}^{T},$$

where ${\tilde{a}}_{m}\left(\varphi ,\theta \right)=e^{j\pi \sin\theta \left(x_{m}\cos\varphi +y_{m}\sin\varphi \right)},\left(x_{m},y_{m}\right)\in \tilde{L}$. In the spectral peak search, the angles corresponding to the peaks are the estimated values $\left({\hat{\varphi }}_{i},{\hat{\theta }}_{i}\right)$.

### 3.3. Unitary-ESPRIT Algorithm

Although 2D-MUSIC can find the DOAs, its total angular field-of-view search is computationally expensive. We construct the URA so that we can introduce the Unitary-ESPRIT [26,27] to give the fast estimations without paring parameters.

First, we define the inverse matrix $\prod_{k}$, whose counter-diagonal values are 1 while the other are 0, as ${\left(\prod_{k}\right)}^{2}={Ι}_{k}$. The unitary matrix is defined to satisfy

(19) $$\prod_{k}Q^{*}=Q$$

Moreover, the matrix can be expressed as

(20) $$Q_{2k}=\frac{1}{\sqrt{2}}\left[\begin{array}{cc} {Ι}_{k} & j{Ι}_{k}\\ \prod_{k} & -j\prod_{k} \end{array}\right]$$

(21) $$Q_{2k+1}=\frac{1}{\sqrt{2}}\left[\begin{array}{ccc} {Ι}_{k} & Ο & j{Ι}_{k}\\ {Ο}^{T} & \sqrt{2} & {Ο}^{T}\\ \Pi_{k} & Ο & -j\Pi_{k} \end{array}\right]$$

The selection matrix is then $J_{1}=\left[\begin{array}{cc} I_{M+N-1} & O \end{array}\right]$ and $J_{2}=\left[\begin{array}{cc} O & I_{M+N-1} \end{array}\right]$. Define

(22) $$K_{1}=Re\left\{Q_{M+N-1}^{H}J_{2}Q_{M+N}^{H}\right\}$$

(23) K2=Im{QM+N−1HJ2QM+NH}

Therefore, we have

(24) $$K_{\mu 1}=I_{M+N}⊗K_{1}$$

(25) $$K_{\mu 2}=I_{M+N}⊗K_{2}$$

(26) $$K_{\nu 1}=K_{1}⊗I_{M+N}$$

(27) $$K_{\nu 2}=K_{2}⊗I_{M+N}$$

Next, we transform $R_{T}$ to give

(28) $$R_{T}=Re\left\{\left(Q_{V}^{H}⊗Q_{V}^{H}\right)R_{C}{\left(Q_{V}^{H}⊗Q_{V}^{H}\right)}^{H}\right\},$$

where $R_{T}$ is a real-valued matrix. The eigenvalue decomposition of a real matrix is less complex than that of a complex matrix, and we can obtain the signal subspace $U_{S}$. Finally, we take the last two steps into consideration and obtain

(29) $$\psi_{\mu }={\left(K_{\mu 1}U_{S}\right)}^{+}K_{\mu 2}U_{S}$$

(30) $$\psi_{\nu }={\left(K_{\nu 1}U_{S}\right)}^{+}K_{\nu 2}U_{S}$$

Define $\psi =\psi_{\mu }+j\psi_{\nu }$ and take the eigenvalue decomposition to acquire the eigenvalue vector λ. We have

(31) $${\hat{\theta }}_{i}=\arcsin\left(\sqrt{{\left(\mu_{i}/\pi \right)}^{2}+{\left(\nu_{i}/\pi \right)}^{2}}\right)$$

(32) $${\hat{\varphi }}_{i}=\arctan\left(\nu_{i}/\mu_{i}\right),$$

where $\left({\hat{\varphi }}_{i},{\hat{\theta }}_{i}\right)$ is the estimated value, $\mu_{i}=2\arctan\left(\left(\lambda_{i}+\lambda_{i}^{*}\right)/2\right)$, and $\nu_{i}=2\arctan\left(\left(\lambda_{i}-\lambda_{i}^{*}\right)/2\right)$. The Unitary-ESPRIT algorithm gives the closed-form solutions of DOAs, thus avoiding the spectral peak search.

### 3.4. Algorithm Steps Conclusion

The main steps of the proposed algorithm can be summarized as follows:

**Step 1**: Calculate the received signals’ covariance matrix ${\overset{\wedge }{R}}_{X}$.
**Step 2**: Given ${\overset{\wedge }{R}}_{X}$, estimate the covariance matrix ${\overset{\wedge }{R}}_{V}$ of the virtual uniform array combined with the array structure.
**Step 3**: Apply the matrix completion theory to estimate the zero-location elements of ${\overset{\wedge }{R}}_{V}$ and obtain ${\overset{}{R}}_{C}$.
**Step 4**: Use the Unitary-ESPRIT algorithm to solve for the estimated DOA values $\left({\hat{\varphi }}_{i},{\hat{\theta }}_{i}\right)$.

## 4. Computational Complexity and Freedom Degree Analysis

### 4.1. Complexity Analysis

**Figure 3:**
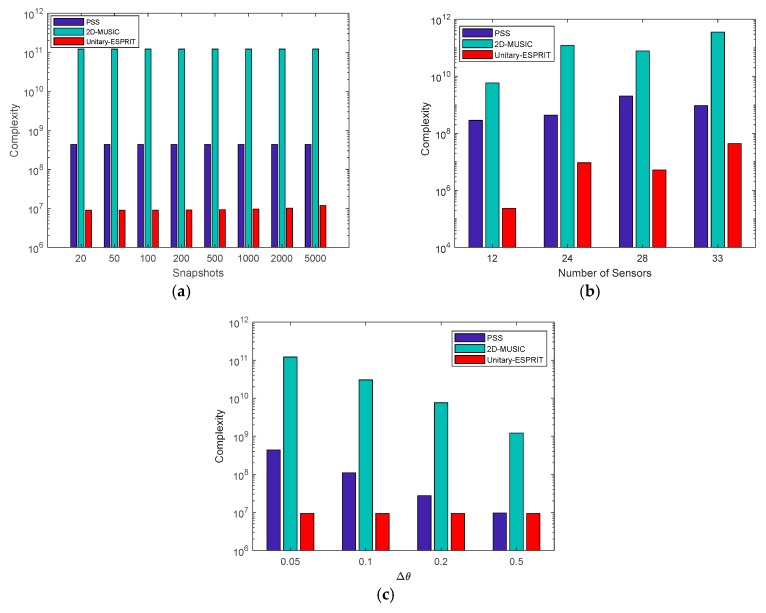
Complexity comparison (**a**) versus snapshots when $M_{1}=3,M_{2}=4,\Delta \theta =0.05^{\circ }$; (**b**) versus the number of sensors when $J=500,\Delta \theta =0.05^{\circ }$; (**c**) versus the searching step when $M_{1}=3,M_{2}=4,J=500$.

The computational complexity of the proposed algorithm using Unitary-ESPRIT is made up of four parts: covariance matrix estimation, SVT, eigenvalue decomposition and Unitary-ESPRIT. The complexities of these four parts are $O\left(JM^{2}\right)$, $O\left(\left(4K\sqrt{K}+8{\left(\sqrt{K}\right)}^{3}\right)N_{t}\right)$, $O\left({\left(V^{2}\right)}^{3}\right)$, and $O\left({\left(2V^{2}\right)}^{3}+K^{3}\right)$, respectively, where $N_{t}$ denotes the iterations number and $V=M_{1}M_{2}-M_{1}+1$. Therefore, the computational complexity of the proposed algorithm is given as $O\left(JM^{2}+\left(4K\sqrt{K}+8{\left(\sqrt{K}\right)}^{3}\right)N_{t}+K^{3}+9V^{6}\right)$. After reconstructing the virtual URA, 2D-MUSIC can also be used to estimate DOAs, and the complexity of the spectral peak search method is $O\left(V^{2}\left(V^{2}-K\right)G_{\varphi }G_{\theta }\right)$, where $G_{\varphi },G_{\theta }$ represent the number of spectral points of the total field-of-view. Hence, the computational complexity of proposed algorithm applying 2D-MUSIC is given as $O\left(JM^{2}+\left(4K\sqrt{K}+8{\left(\sqrt{K}\right)}^{3}\right)N_{t}+V^{6}+V^{2}\left(V^{2}-K\right)G_{\varphi }G_{\theta }\right)$. Moreover, the complexity of the PSS method [15] is $O\left(J\left(M_{1}^{4}+M_{2}^{4}\right)+M_{1}^{6}+M_{2}^{6}+G_{\varphi }G_{\theta }\left(\frac{M_{1}^{4}}{M_{2}^{2}}+\frac{M_{2}^{4}}{M_{1}^{2}}\right)\right)$. For the sake of clarity, the computational complexity of all these methods is summarized in Table 1. We also compare the complexity of methods versus snapshots (J), the number of sensors (M) and the searching step (Δθ=Δφ, where $G_{\theta }=90/\Delta \theta ,G_{\varphi }=360/\Delta \varphi$) in Figure 3a–c, respectively.

As shown in Figure 3, Unitary-ESPRIT has the smallest complexity. The number of snapshots has a weak impact on complexity, while the searching step has the strongest impact. The PSS method and the proposed algorithm using 2D-MUSIC are both based on a 2D search, which means that the complexity is high when the searching step is small. Therefore, we introduce the Unitary-ESPRIT algorithm to replace the search methods and provide the closed-form solutions for the DOAs. Hence, the searching step does not affect the complexity of the proposed algorithm using Unitary-ESPRIT. The main component of the computational complexity in the proposed algorithm is SVT, where the iteration number is, on average, 200. However, compared with the search methods, the complexity is relatively low. As a result, the proposed algorithm can reduce complexity to realize fast estimations.

### 4.2. Freedom Degree Analysis

**Figure 4:**
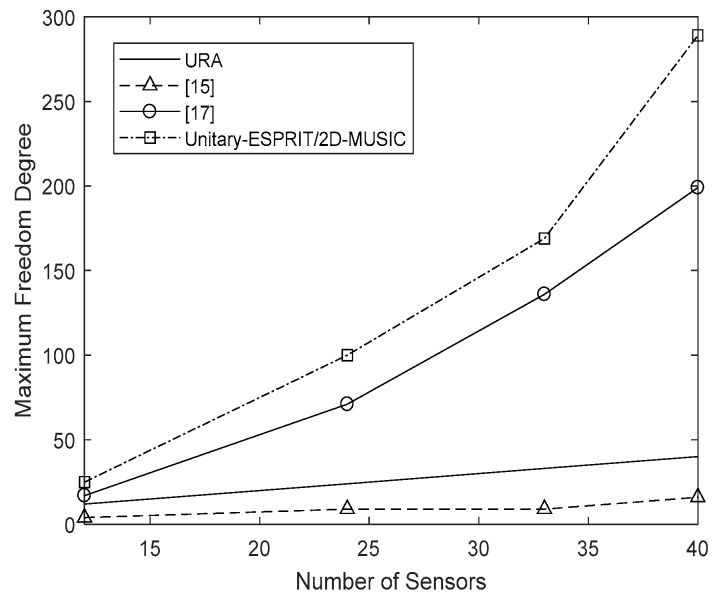
Freedom degree versus the number of sensors.

The degree of freedom is affected by the array and the algorithm that we choose, and determines the maximum number of signals that we can estimate directly. The algorithms presented in [15] and [17] have freedom degrees of $O\left(M_{1}^{2}-1\right)$ and $O\left(\left(M^{2}-1\right)/8\right)$, respectively. The proposed algorithm reconstructs a V×V virtual URA based on the coprime planar array with the M sensors number. The Unitary-ESPRIT or 2D-MUSIC used to estimate DOAs has no influence to degree of freedom, and each one obtains a degree of freedom of $O\left(V^{2}-1\right)$. The detailed values are presented in Figure 4. The freedom degree of the algorithm in [15] depends on $M_{1}$, so it is the lowest, while that of the URA is no more than the number of sensors. Both the proposed algorithm and the method introduced in [17] have freedom degrees that are greater than the number of sensors. Comparing the two algorithms, we find that the proposed algorithm has a higher degree of freedom. Thus, we have developed an algorithm using coprime planar arrays that offers a high degree of freedom, enabling multiple signals to be resolved.

## 5. Simulation Results

This section reports the results of performance simulation experiments comparing the proposed algorithm using Unitary-ESPRIT with that using 2D-MUSIC [25] and the PSS method [15]. To measure the accuracy of the algorithms, we define the root mean square error (RMSE) as

(33) $$\mathrm{RMSE}=\sqrt{\frac{1}{QK}\sum_{i=1}^{Q}{\left\|\gamma -{\hat{\gamma }}_{i}\right\|}^{2}}$$

where Q denotes the number of simulation; γ and ${\hat{\gamma }}_{i}$ are defined as the real values and the ith estimated values, respectively. We assume that $M_{1}=3,M_{2}=4$, and then have *M* = 24, *V* = 10.

**Simulation** **1:** Performance under the condition of multiple signals (*K* > *M*).

**Figure 5:**
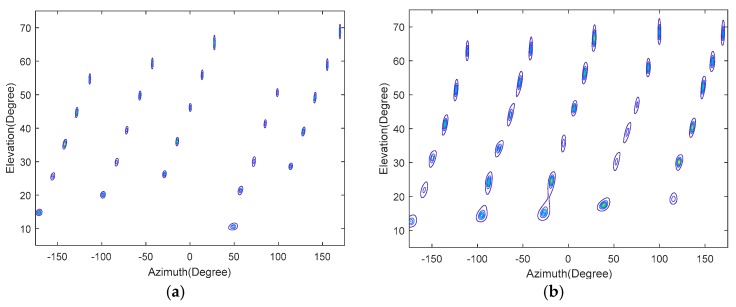
Spatial spectrum contour map (**a**) K=25 (**b**) K=30.

**Figure 6:**
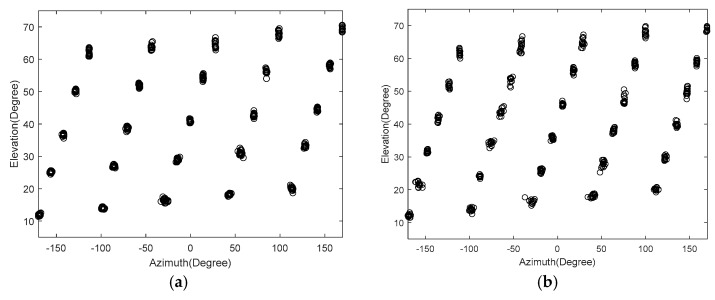
Distribution of azimuth and elevation estimation (**a**) K=25 (**b**) K=30.

We consider the cases of K=25 or K=30 signals with a signal-to-noise ratio (SNR) of 10 dB,J=500 and $\Delta \theta =\Delta \varphi =0.05^{\circ }$. The spatial spectrum contour map of the total field-of-view is shown in Figure 5, as given by 2D-MUSIC algorithm. The distribution of the estimated values from 20 simulations using the Unitary-ESPRIT algorithm is presented in Figure 6. Figure 5 shows that we can find the 25 or 30 peaks, and proves that the proposed method using 2D-MUSIC can solve problems in which the number of signals is greater than the number of sensors. Moreover, Figure 6 shows that the proposed algorithm using Unitary-ESPRIT gives estimated values that are close to the real values over 20 simulations, indicating robust performance. Compared with the PSS method, which finds at most $M_{1}^{2}$ signals, we make better use of the high freedom degree to estimate more signal directions.

**Simulation** **2:** RMSE comparison of the proposed algorithm using Unitary-ESPRIT and 2D-MUSIC, the PSS method, and the URA with the same number of sensors under different SNRs.

**Figure 7:**
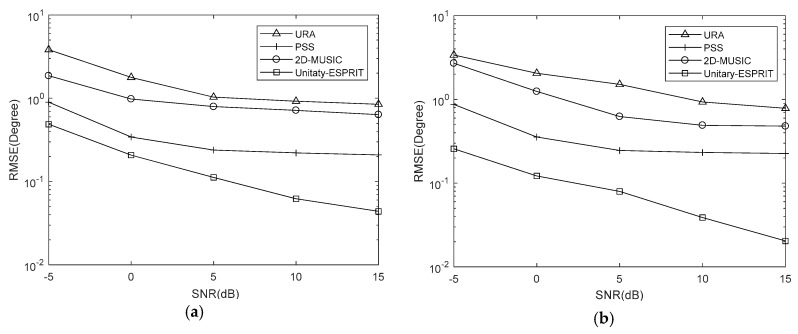
RMSE comparison under different SNRs with $K=7,\Delta \theta =\Delta \varphi =0.5^{\circ }$ (**a**) azimuth (**b**) elevation.

**Figure 8:**
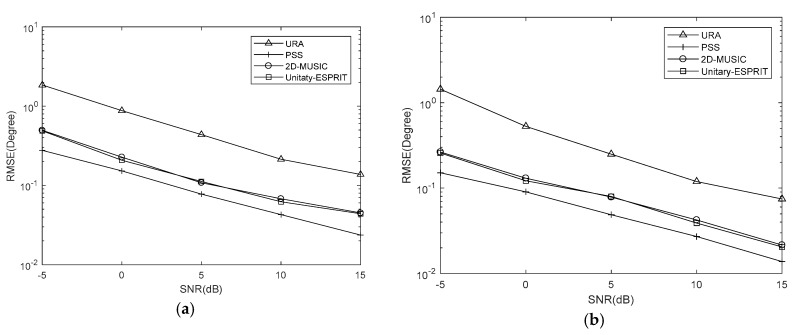
RMSE comparison under different SNRs with $K=7,\Delta \theta =\Delta \varphi =0.05^{\circ }$ (**a**) azimuth (**b**) elevation.

**Figure 9:**
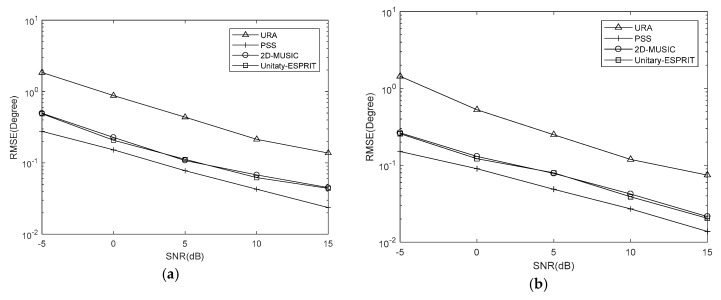
RMSE comparison under different SNRs with $K=10,\Delta \theta =\Delta \varphi =0.05^{\circ }$ (**a**) azimuth (**b**) elevation.

Simulations are conducted with Q=100, J=500 and SNRs from −5 dB to 15 dB at 5 dB intervals. And we set $K=7,\Delta \theta =\Delta \varphi =0.5^{\circ }$, $K=7,\Delta \theta =\Delta \varphi =0.05^{\circ }$ and $K=10,\Delta \theta =\Delta \varphi =0.05^{\circ }$. The RMSE results are shown in Figure 7, Figure 8 and Figure 9, respectively. 

It is evident that the RMSE of the coprime planar array is lower than that of URA with the same number of sensors. This is because the coprime planar array has a bigger array aperture when the number of sensors is known. The RMSEs of the proposed algorithm using 2D-MUSIC and PSS are affected by the searching step, while that of proposed algorithm using Unitary-ESPRIT are not. The former two methods can obtain the lower RMSE when the searching step is smaller. The results verify that Unitary-ESPRIT has lower RMSE than 2D-MUSIC when $\Delta \theta =\Delta \varphi =0.5^{\circ }$. But 2D-MUSIC gives a similar RMSE to Unitary-ESPRIT when $\Delta \theta =\Delta \varphi =0.05^{\circ }$. Given the results of our complexity analysis, 2D-MUSIC obtains similarly precise estimations as Unitary-ESPRIT at the cost of higher complexity. 

When $K=7,\Delta \theta =\Delta \varphi =0.05^{\circ }$, the PSS method is effective and outperforms the proposed algorithm using Unitary-ESPRIT. There are two reasons for this. One is the searching step of PSS is small. The other is the covariance matrix of virtual URA is estimated by SVT, which introduces errors to the covariance matrix. But the PSS calculates the covariance matrix of coprime planar array directly, without additional methods. However, when $K=7,\Delta \theta =\Delta \varphi =0.5^{\circ }$ and $K=10,\Delta \theta =\Delta \varphi =0.05^{\circ }$, the PSS method has the higher RMSE than proposed algorithm using Unitary-ESPRIT, and fails; however, Unitary-ESPRIT maintains its accuracy. Although the PSS can have lowest RMSE, it may come at the cost of higher complexity. And when applied to multiple signals, the proposed algorithm using Unitary-ESPRIT is preferable and offers real-time performance due to the minimal complexity.

**Simulation** **3:** RMSE comparison of the proposed algorithm using Unitary-ESPRIT and 2D-MUSIC, the PSS method and the URA with the same number of sensors under different number of snapshots.

**Figure 10:**
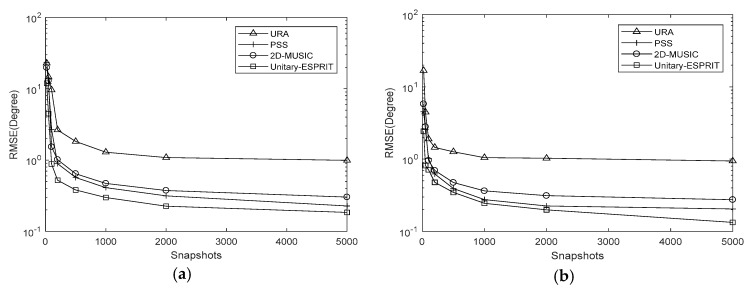
RMSE comparison under different snapshots with $K=7,\Delta \theta =\Delta \varphi =0.5^{\circ }$ (**a**) azimuth (**b**) elevation.

**Figure 11:**
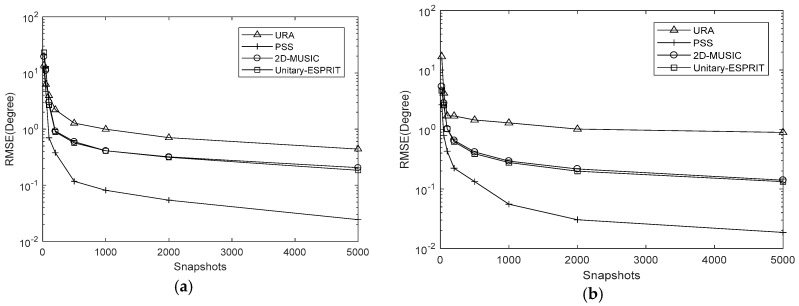
RMSE comparison under different snapshots with $K=7,\Delta \theta =\Delta \varphi =0.05^{\circ }$ (**a**) azimuth (**b**) elevation.

**Figure 12:**
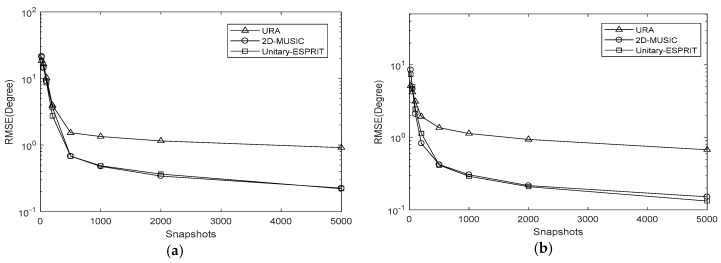
RMSE comparison under different snapshots with $K=10,\Delta \theta =\Delta \varphi =0.05^{\circ }$ (**a**) azimuth (**b**) elevation.

In simulation 3, we set the SNR to 15 dB and varied the number of snapshots to J=[20,50,100,200,500,1000,2000,5000]. The results are presented in Figure 10, Figure 11 and Figure 12. The RMSE decreases as the number of snapshots increases, although the decline is negligible once J>500. The conclusions of simulation 2 are also applicable to the number of snapshots.

## 6. Conclusions

This paper has presented a DOA estimation technique for multiple signals using a coprime planar array. Using matrix completion theory combined with the Unitary-ESPRIT algorithm, the proposed technique overcomes the problems of existing algorithms, namely the high computational complexity of the 2D spectral peak search and an inability to find more signals than the number of sensors. This paper has described the model and the associated algorithm, and analyzed the computational complexity of the proposed algorithm in comparison with that of existing algorithms. Through theoretical analysis and a series of simulation experiments, we can conclude that the proposed algorithm attains higher accuracy than URA with the same number of sensors. Using the matrix completion theory to estimate the covariance matrix of a virtual URA, we can obtain a better degree of freedom to find multiple signals. Furthermore, applying the Unitary-ESPRIT algorithm to find the closed-form DOA solution greatly reduces the computational complexity.

## Figures and Tables

**Table 1:** Computational Complexity Comparison of Different Algorithms.

Algorithms	Complexity
Unitary-ESPRIT	$O\left(JM^{2}+12K\sqrt{K}N_{t}+K^{3}+9V^{6}\right)$
2D-MUSIC	$O\left(JM^{2}+12K\sqrt{K}N_{t}+V^{6}+V^{2}\left(V^{2}-K\right)G_{\varphi }G_{\theta }\right)$
PSS	$O\left(J\left(M_{1}^{4}+M_{2}^{4}\right)+M_{1}^{6}+M_{2}^{6}+G_{\varphi }G_{\theta }\left(\frac{M_{1}^{4}}{M_{2}^{2}}+\frac{M_{2}^{4}}{M_{1}^{2}}\right)\right)$
